# Fecal microbiota transplantation in adolescents with obesity induces limited and donor-dependent remodeling of the gut microbiome

**DOI:** 10.3389/fendo.2026.1927870

**Published:** 2026-09-09

**Authors:** Xinchao Jiang, Shuqi Shi, Min Li, Yue Zhang, Guorui Xing, Qiulong Yan, Shenghui Li, Wuping Sun, Haiyong Li

**Affiliations:** 1 Department of Pain Medicine, Suzhou Hospital of Integrated Traditional Chinese and Western Medicine, Suzhou, Jiangsu, China; 2 Department of Clinical Laboratory Medicine, Fifth Affiliated Hospital, Southern Medical University, Guangzhou, China; 3 Puensum Genetech Institute, Wuhan, China; 4 Center for Microbiome Medicine, The Fifth Affiliated Hospital, Southern Medical University, Guangzhou, China

**Keywords:** adolescents, fecal microbiota transplantation (FMT), gut microbiome, metagenome, obesity

## Abstract

**Introduction:**

Obesity is a global health challenge, and fecal microbiota transplantation (FMT) is considered a potential intervention; however, its long-term ecological impact remains unclear.

**Methods:**

In this 182-day longitudinal cohort of adolescents with obesity (378 samples), metagenomic sequencing was used to assess FMT-induced remodeling of the multi-kingdom gut ecosystem through species composition, diversity, microbial networks, and machine learning analyses.

**Results:**

FMT induced only transient shifts in community structure, with limited donor strain engraftment and strong resilience of recipient core taxa. Bacteria were the primary responders, the virome showed short-term perturbation, and network restoration was partial and donor-dependent.

**Discussion:**

FMT exerts limited, donor-dependent ecological effects in obese adolescents. Optimizing donor selection and personalized matching may be essential for improving long-term efficacy.

## Graphical Abstract

**Figure:**
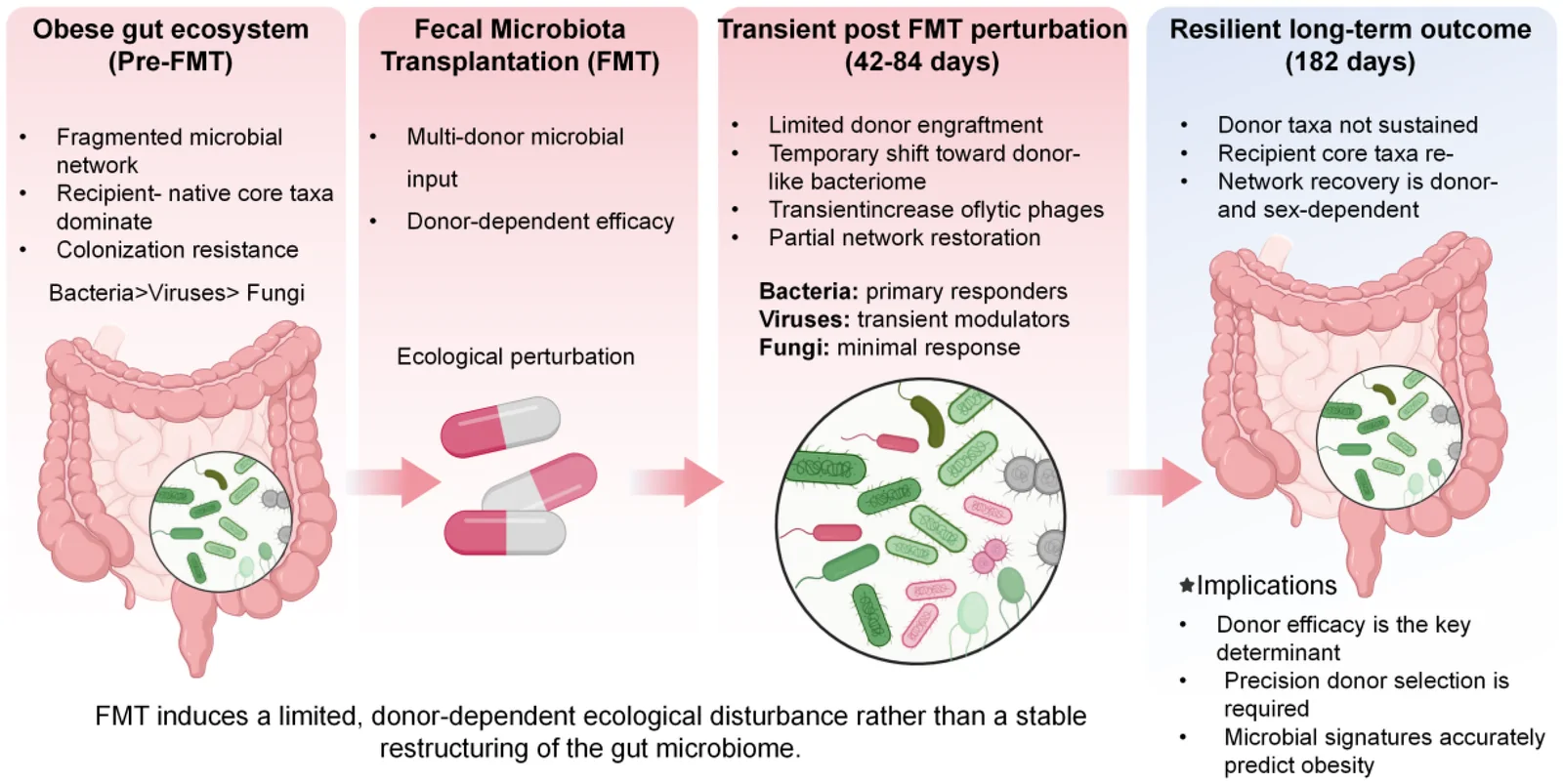
Donor-dependent and transient remodeling of the gut microbiome following fecal microbiota transplantation (FMT) in adolescents with obesity.

## Highlights

- Donor engraftment after FMT is limited and transient, while recipient core taxa remain resilient.
- Microbiome responses to FMT are highly donor-dependent, consistent with a “super-donor” effect.
- FMT partly restores obesity-disrupted microbial networks, and key microbial features accurately classify obesity.

## 1 Introduction

The global prevalence of overweight and obesity is rising at an unprecedented rate (1). Currently, an estimated 33% of adults worldwide are classified as overweight (BMI 25–30 kg/m²) or obese (BMI >30 kg/m²), a figure expected to increase in the coming years (2). Obesity is not merely a disorder of excess body weight but also a critical driver of numerous serious comorbidities, including hypertension, type 2 diabetes, cardiovascular diseases, among others (3). Given that childhood and adolescent obesity exhibits a high propensity to persist into adulthood and substantially elevates the risk of morbidity and mortality in later life, early intervention is essential to disrupt this disease trajectory and improve long-term health outcomes (4).

While lifestyle interventions, pharmacological treatments, and even bariatric surgery have been employed in the management of obesity, these strategies are often limited by poor adherence, safety concerns, or limited accessibility in adolescent populations, and their long-term efficacy is frequently suboptimal (5). Consequently, there is a pressing need to develop novel, safe, and sustainable early intervention strategies to fundamentally reshape the pathophysiological trajectory of obesity. In recent years, the gut microbiota has been identified as a key mediator regulating host energy balance, appetite control, inflammatory responses, and glucose and lipid metabolism (6, 7). Research confirms that the gut microbiota of individuals with obesity exhibits characteristic dysbiosis, typically featuring a reduced abundance of short-chain fatty acid-producing commensal bacteria (such as *Faecalibacterium* and *Roseburia*) alongside a relative enrichment of potentially pro-inflammatory bacteria (e.g., certain species of *Bacteroides* and *Prevotella*) (8). In this context, fecal microbiota transplantation (FMT) has been proposed as a promising therapeutic avenue. It aims to alter the energy intake increase associated with obesity and modulate the gut microbial community by suppressing harmful microbiota and restoring metabolic homeostasis (9, 10).

Transplantation of fecal microbiota from obese donors into germ-free mice has been shown to induce weight gain, fat accumulation, and insulin resistance, whereas FMT from lean donors confers protective effects (11–15). Inspired by these findings, several clinical trials have explored lean-donor FMT in adults with obesity or metabolic syndrome. Results indicate that FMT can yield short-term improvements in insulin sensitivity (16–19), enhance gut barrier function (20), and ameliorate markers of glucose metabolism (21, 22). However, to date, no high-quality randomized controlled trial has conclusively demonstrated that FMT effectively reduces body weight or fat mass, leaving its clinical value as a weight-loss therapy a subject of debate. In a previous double-blind randomized placebo-controlled trial involving 87 obese adolescents, it was also found that encapsulated FMT had no significant effect on BMI SDS or total body fat percentage over six months (23, 24). Notably, in participants with concomitant metabolic syndrome, a decrease in visceral fat proportion and temporary improvement in insulin resistance were observed, accompanied by sustained remodeling of receptor microbiome composition and function (24–26). These divergent outcomes suggest that the therapeutic potential of FMT may be contingent on specific host factors and requires a deeper understanding of the underlying microbial ecology.

Crucially, most prior FMT research has centered on bacteria or, more recently, the virome in isolation. The longitudinal dynamics of multi-kingdom communities (bacteria, fungi, viruses) post-FMT, their cross-kingdom interactions, and how these collective changes relate to sustained metabolic outcomes remain largely unexplored. To bridge this gap, we leveraged the long-term follow-up cohort from the aforementioned adolescent trial (24). By integrating longitudinal metagenomic data across all three microbial kingdoms, this study aimed to characterize the nature and extent of gut ecosystem perturbation induced by FMT, to elucidate the respective roles of donor characteristics and recipient factors in shaping community recovery, and to assess the ecosystem-level structural changes associated with intervention.

## 2 Materials and methods

### 2.1 Data collection

We obtained a dataset of 381 fecal metagenomes from a study on obesity by Wilson et al. (24) (BioProject: PRJNA637785) via the NCBI Sequence Read Archive (SRA) (**Supplementary Table 1**). This study included 87 post-pubertal adolescents with obesity (51 females, 36 males) and 9 donors (4 females, 5 males). Participants were stratified by sex and randomly assigned (1:1) to receive either active multi-donor fecal microbiota transplantation (FMT) or a saline placebo, administered orally using 28 acid-resistant capsules over two consecutive days. Each FMT recipient received capsules derived from four same-sex donors, with a total of 28 capsules given: 16 (4 from each donor) on day 1, followed by 12 (3 from each donor) on day 2. To maintain intervention consistency, the same donor set was used throughout the trial, except for one male donor (DM05) who was replaced (by DM12) after the first treatment round due to illness. Fecal samples were collected and clinical assessments were conducted at pre-FMT and at 42, 84, and 182 days post-treatment (24). The parent randomized controlled trial was registered with the Australian New Zealand Clinical Trials Registry (ACTRN12615001351505) and approved by the Northern A Health and Disability Ethics Committee (16/NTA/172), with the 4-year follow-up extension conducted under amendment 16/NTA/172/AM08. For the current analysis, three participants (one female, two males) who provided only pre-FMT samples were excluded. This study generated approximately 1.93 Tb of clean data from 378 samples (**Supplementary Table 1**).

### 2.2 Data preprocessing

Raw reads were quality-controlled via fastp v0.23.4 (27) with the options ‘-q 20 -u 30 -n 5 -y -Y 30 --trim_poly_g --trim_poly_x -j’ and bbduk (28) with the parameters ‘entropy=0.6 entropywindow=50 entropyk=5’. Furthermore, reads from human and *Escherichia phage phiX174* were removed by matching quality-filtered reads against the CHM13v2.0 and phiX174 (NCBI accession NC_001422.1) genomes with bowtie2 v2.4.4 (29).

### 2.3 Taxonomic profiling and diversity

Quality-controlled reads were taxonomically classified using Kraken2 (v2.1.3) against our custom iBFV database, with the confidence threshold set to 0.1 as previously optimized (30). Species-level relative abundance estimates were derived through Bayesian re-estimation using Bracken v2.8 (31). Key parameters included: read length specification (150 bp matching sequencing fragments), minimum read threshold (5 reads), and taxonomic level restriction to species rank (-l S).

### 2.4 Definition of donor batch efficacy

To classify donor batches by their capacity to induce sustained microbial convergence, we calculated the Bray-Curtis distance between each post-FMT recipient sample and the centroid of their respective four-donor pool (i.e., the average species-level profile of the four same-sex donors) at days 42, 84, and 182. A donor batch was classified as “high-efficacy” if its recipients exhibited: (1) a significantly lower median distance to the donor pool centroid at day 182 compared to other batches of the same sex (Wilcoxon rank-sum test, *p* < 0.05); and (2) sustained convergence, defined as a maintained reduction in distance from pre-FMT levels across all time points without significant reversion toward baseline. Batches not meeting these criteria were classified as “low-efficacy.” This classification was performed separately for females and males.

### 2.5 Identification of the biomarkers

To identify potential microbial biomarkers, we first filtered microbial features with a relative abundance greater than 0.01% across different groups. This yielded 900 microbial features in female samples and 880 in male samples for downstream analysis. Using Multivariate Association with Linear Models (MaAsLin2) (32), we compared species-level abundance between donor samples and pre-FMT samples. This analysis identified 411 microbial features in females and 229 in males that showed differential abundance between donor and pre-FMT groups. These differentially abundant features were selected as candidate biomarkers.

### 2.6 Correlation network analysis

Use spearman correlation analysis to evaluate the co-occurrence relationship between paired bacteria and viruses, and adjust the p-value using the function *p.adjust* with the option “method=BH” in the R platform. The relevant network shows an absolute spearman coefficient > 0.40 and a q-value < 0.05.

### 2.7 Random forest analysis

Random forest classification was implemented using the randomForest::randomForest function in R (v4.3.0) with a 10-times repeated threefold cross-validation strategy. To strictly prevent data leakage, all feature selection and model training were conducted exclusively within each cross-validation fold using only the training data. Specifically, within each fold, features were ranked by their variable importance scores (mean decrease in Gini index) derived from the training set, and the top *N* features were retained for model construction. This internal feature selection process was independently repeated for each fold, ensuring complete isolation of test set information. The entire procedure was iterated 10 times with different random seeds to ensure robustness of the performance estimates.

Given that the dataset includes repeated measurements from the same individuals across multiple time points, a subject-level splitting strategy was strictly applied. All samples originating from the same subject were exclusively assigned to either the training or test set within each cross-validation fold, preventing samples from the same individual from appearing across both sets. This design effectively ensures that the model captures genuine obesity-associated microbial signals rather than subject-specific fingerprints.

Model performance was evaluated using the area under the receiver operating characteristic curve (AUC), as well as accuracy, sensitivity, and specificity, calculated via the *pROC::roc* function. The relative contribution of each feature to the classification was quantified by the mean decrease in Gini index, and the top-ranking discriminatory taxa were visualized via importance plots.

### 2.8 Statistical methods

All statistical analyses were performed in R (v4.3.1) environment. Alpha diversity was assessed by calculating observed richness and the Shannon diversity index from species-level relative abundance profiles. Between-group differences in alpha diversity indexes were quantified as Hedges’ g standardized mean differences using the *metafor::escalc* and *metafor::rma* functions. In addition, Bray-Curtis distances between samples were computed based on the species-level relative abundance profile using the *vegan::vegdist* function (33). Using this distance matrix, we conducted a permutational multivariate analysis (PERMANOVA) using the *vegan::adonis* function. The obtained R² values were further adjusted using the *RsquareAdj* function. Similarly, based on the same distance matrix, principal coordinate analysis (PCoA) was carried out using the *ape::pcoa* function. The PCoA plot was generated using the *ggplot* function.

## 3 Results

### 3.1 Sequencing data and cohort characteristics

A total of 378 samples were included in the final analysis. The cohort was stratified by sex, with separate donor and recipient groups for females and males. In the female cohort, samples comprised 34 from 4 donors, 39 from adolescents with obesity at baseline, 69 from the FMT group across three post-intervention time points (24 at day 42, 23 at day 84, and 22 at day 182), and 26 from the placebo group at baseline plus 72 across the corresponding follow-up time points (26, 23, and 23, respectively). In the male cohort, samples included 24 from 5 donors, 15 from adolescents with obesity at baseline, 43 from the FMT group (15 at day 42, 14 at day 84, and 14 at day 182), and 19 from the placebo group at baseline plus 52 across the same follow-up time points (18, 17, and 17, respectively). Detailed sample information was provided in **Supplementary Table 1**.

### 3.2 FMT leads to limited donor engraftment and transient community perturbation

**Figure 1:**
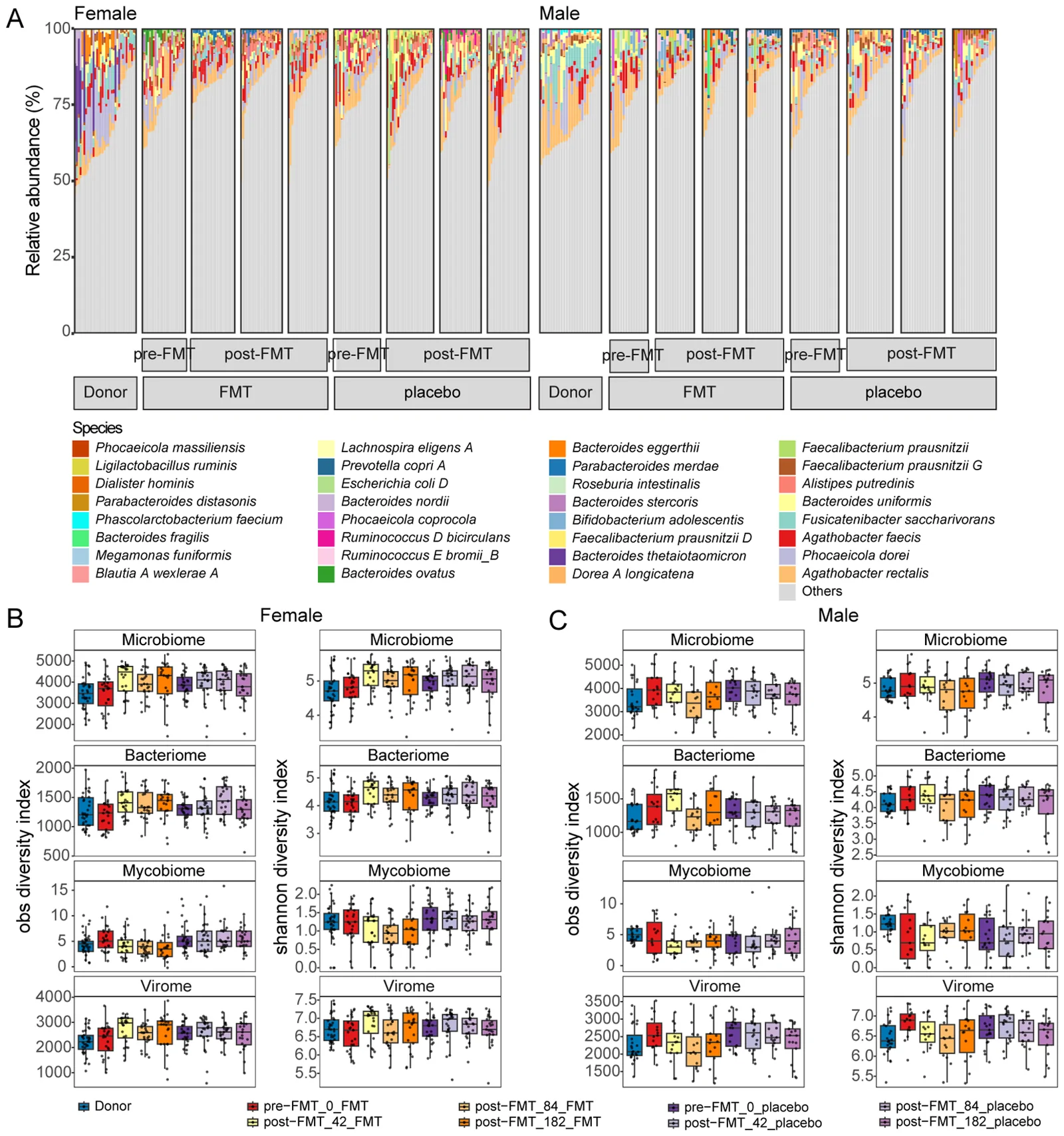
Microbial composition and diversity dynamics following FMT in obese adolescents by sex. **(A)** Longitudinal changes in relative abundance of major bacterial species across donor, FMT recipient (female and male), and placebo groups. Data are shown as stacked bar plots representing the percentage composition of each taxon at donor, pre-FMT and post-FMT time points (0, 42, 84, and 182 days). **(B, C)** Changes in microbial diversity indices over time in female **(B)** and male **(C)** participants. Box plots show the observed diversity index (obs diversity) and Shannon index for the total microbiome (Microbiome), bacteriome (Bacteriome), mycobiome (Mycobiome), and virome (Virome) at donors and pre-FMT and multiple post FMT time points (0, 42, 84, and 182 days) in the FMT and placebo groups.

We evaluated the engraftment of donor-derived bacteria and the resulting changes in microbial community structure following FMT (**Figure 1A**). Species-level analysis revealed limited colonization by signature donor taxa. Instead, recipient-native species, particularly *Agathobacter rectalis*, remained predominant. The relative abundance of *A. rectalis* displayed notable temporal dynamics, showing a transient decrease in FMT recipients at 42 days (3.54% in females; 3.12% in males) compared to their pre-FMT and placebo controls, followed by recovery towards pre-FMT levels by day 182. This pattern indicates that FMT induced an early, transient perturbation in the gut ecosystem, but the microbiota exhibited strong resilience over the 182-day observation period.

Alpha-diversity analysis across the entire microbial community (bacteria, fungi, viruses) revealed no significant global shifts (**Figure 1B**). Female and male FMT recipients showed a transient, non-significant increase in Shannon diversity post-FMT. Fungal diversity was consistently low and unaffected by FMT. Viral diversity remained high with no significant treatment-induced changes, except for a modest, delayed increase in Shannon index among female recipients at day 182.

### 3.3 Microbial community shifts exhibit significant donor-dependence

Donor-dependent effects on bacterial diversity were evident in both female and male recipients (**Supplementary Figure 1**). Specifically, recipients of the DF16-containing donor batch (females) and the DM07/DM08-containing donor batches (males) achieved higher post-FMT alpha diversity compared to recipients of other donor batches within their respective cohorts, underscoring that donor selection, rather than recipient sex, may critically influences the structural outcome.

**Figure 2:**
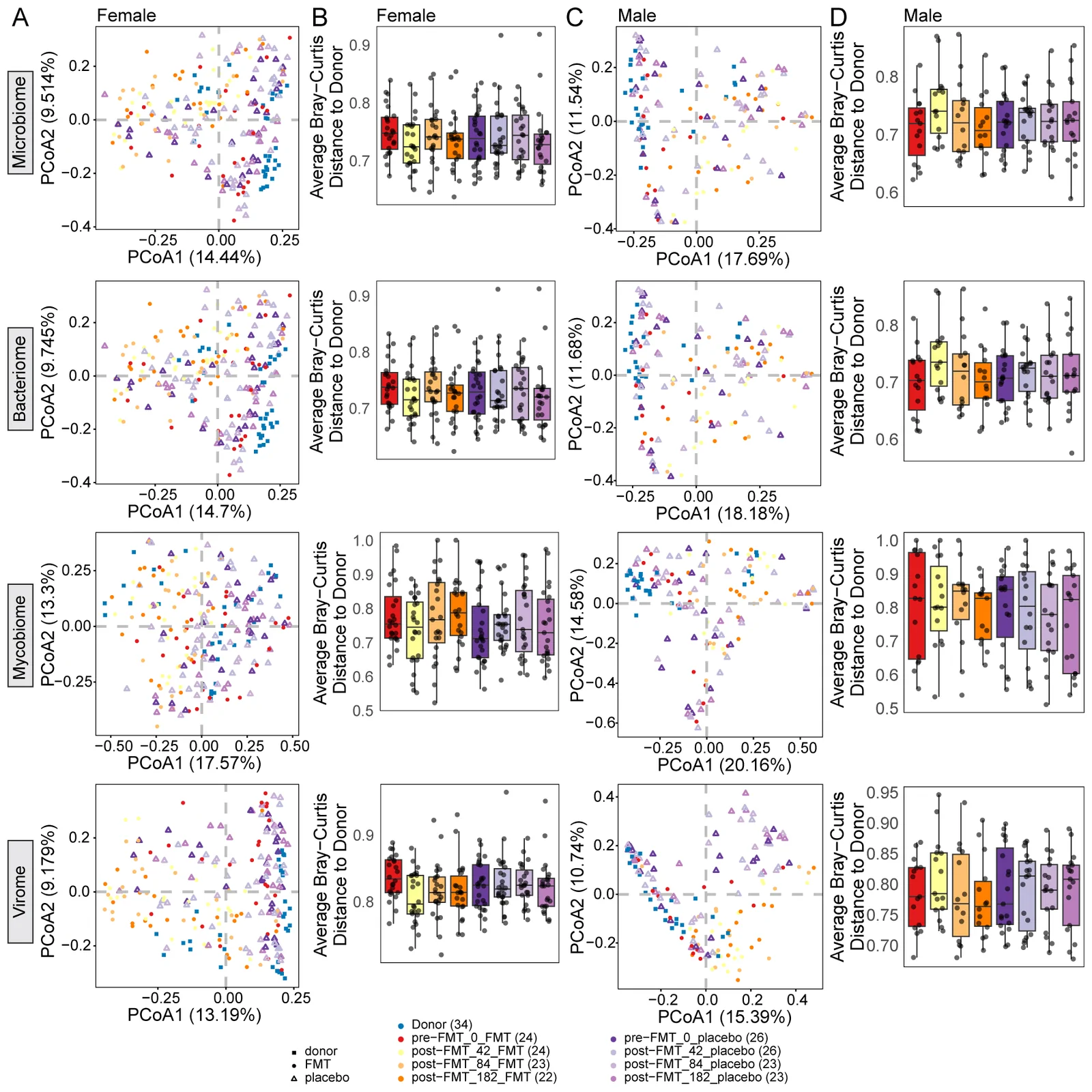
Microbial community structure and donor similarity dynamics following FMT in obese adolescents by sex. **(A, C)** Principal coordinate analysis (PCoA) plots based on Bray-Curtis dissimilarity matrices showing the beta-diversity of the microbiome across different time points and treatment groups in female **(A)** and male **(C)** participants. Each panel represents one microbial domain: total microbiome, bacteriome, mycobiome, and virome. **(B, D)** Box plots showing the average Bray-Curtis distance to the donor microbiome for each microbial domain in female **(B)** and male **(D)** participants across time points. Lower distances indicate greater similarity to the donor profile.

Analysis of Bray-Curtis distances at the group level demonstrated that FMT transiently shifted the gut bacterial community of recipients toward a donor-like structure (**Figure 2**). In female recipients, the bacteriome progressively converged with the donor profile from day 42 post-FMT and remained stably close to it throughout the 182-day follow-up. Male recipients exhibited only a transient convergence at day 42, followed by gradual reversion to a pre-FMT-like state by day 182. However, the degree and sustainability of convergence appeared to be primarily donor-dependent. Recipients of high-efficacy donor batches (e.g., the DF16-containing female batch, the DM08-containing male batch) maintained closer and more stable similarity to their respective donor pool centroids, whereas recipients of low-efficacy donor batches showed limited or transient convergence (**Supplementary Figures 2, S3**). No such directional shifts occurred in placebo groups, suggesting FMT-specific effects. Fungal and viral communities showed minimal responses across both cohorts, indicating bacteria as the primary target.

### 3.4 Long-term efficacy of FMT is determined by donor efficacy and modulated by host factors

FMT triggered a transient ecological transition of the viral community across genders, with temperate phages dominating the pre-FMT, accounting for approximately 26.87% of phages in females and 26.77% in males (**Supplementary Figure 4**). By day 84 post-FMT, the proportion of lytic phages increased in both females (from 28.26% to 29.93%) and males (from 26.71% to 31.28%), with a corresponding decrease in temperate phages, followed by a partial recovery by day 182. No such changes occurred in placebo groups, indicating an FMT-specific, short-term perturbation toward increased lytic phage activity.

**Figure 3:**
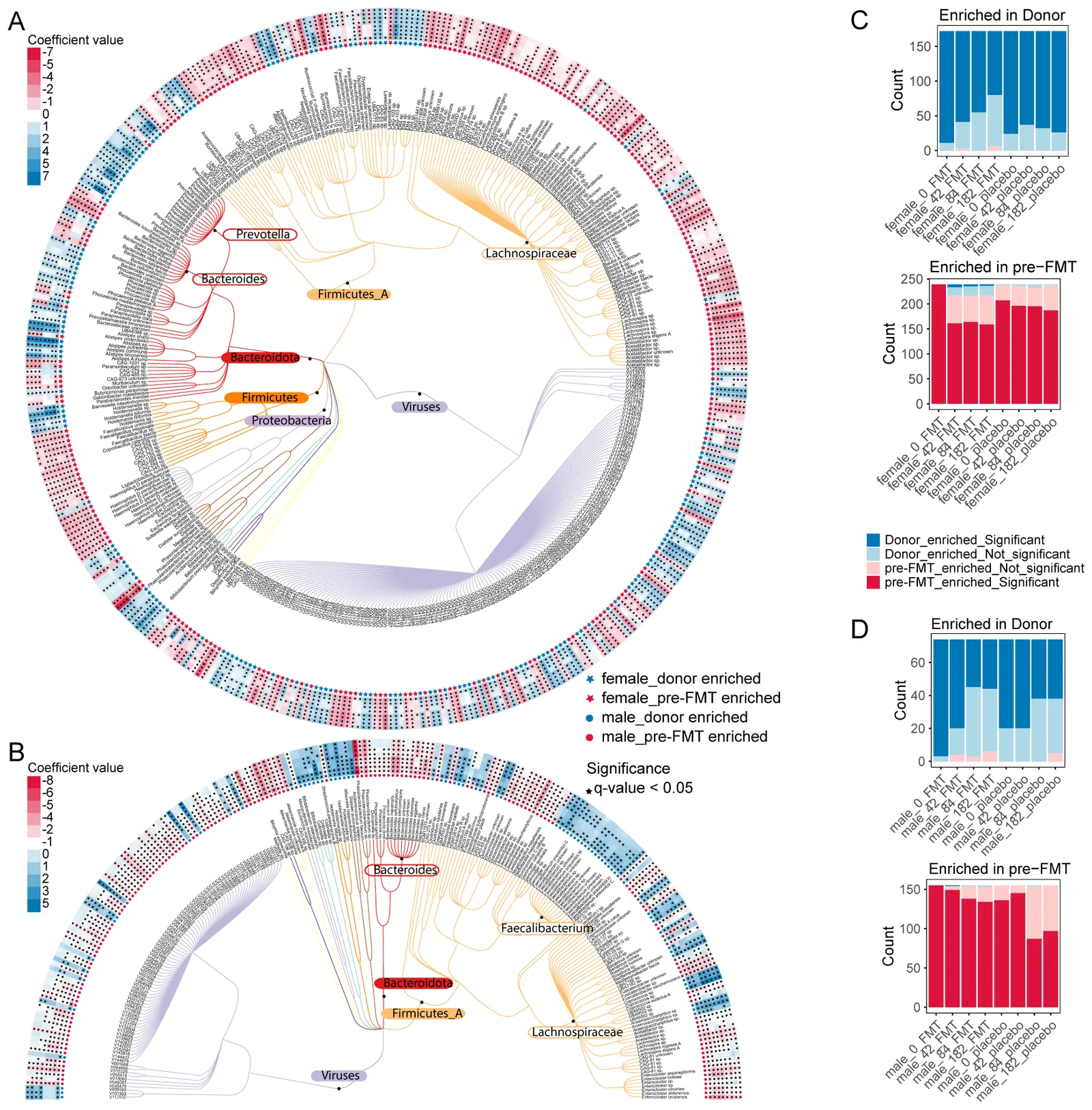
Taxonomic enrichment and phylogenetic distribution of microbial communities in donors and pre-FMT recipients across sexes. **(A)** Phylogenetic tree and longitudinal differential abundance of bacterial and viral taxa in female participants. The innermost ring displays a maximum-likelihood phylogenetic tree constructed from representative OTUs/ASVs, with major phyla (e.g., *Firmicutes*, *Bacteroidetes*, *Proteobacteria*) and key families (e.g., *Lachnospiraceae*, *Ruminococcaceae*) labeled. Surrounding rings represent coefficient values from linear mixed-effects models comparing each taxon’s relative abundance against the donor at sequential time points: female_pre-FMT_0_FMT, post-FMT_42_FMT, 84_FMT, 182_FMT, and corresponding placebo groups (0_placebo, 42_placebo, etc.). Color intensity and direction indicate magnitude and direction of enrichment: blue denotes enrichment in the donor (negative coefficient), while red indicates enrichment in the recipient (positive coefficient). Statistically significant differences (q-value < 0.05) are marked with a star. Dots outside the tree denote species enriched in either female or male donors or pre-FMT recipients, with color-coded legend indicating source. **(B)** Same as panel A but for male participants. **(C)** Number of microbial taxa significantly enriched in the donor across time points and treatment groups in females. Colors distinguish whether enrichment is significant (dark blue) or not (light blue) in the donor group. Red and pink indicate enrichment in FMT recipients (significant or not). **(D)** Same as panel C but for males.

Differential abundance analysis tracked the post-intervention evolution of microbial signatures (**Figure 3**; **Supplementary Tables 2, 3**). In female FMT recipients (**Figure 3A**; **Supplementary Table 2**), donor-enriched taxa were primarily represented by *Prevotella*. In contrast, recipient-enriched taxa before FMT included various members of the *Lachnospiraceae*. Following FMT (**Figure 3C**), donor-enriched signatures gradually declined over time, whereas recipient-enriched signatures—which were most abundant at pre-FMT—showed limited change during the follow-up period. No clear directional shifts in either type of signature were observed in the female placebo group.

In male FMT recipients (**Figure 3B**; **Supplementary Table 3**), donor-enriched taxa were predominantly from the genus *Faecalibacterium*. In contrast, pre-FMT enriched taxa consisted of various *Bacteroides* and *Lachnospiraceae* species. Following FMT (**Figure 3D**), both donor- and recipient-enriched signatures gradually decreased and remained relatively stable from day 84 to day 182. In the male placebo group, only minor changes occurred between pre-FMT and day 42, with more pronounced variations observed thereafter.

### 3.5 Microbial co-occurrence network structure is remodeled in a donor-dependent manner following FMT

**Figure 4:**
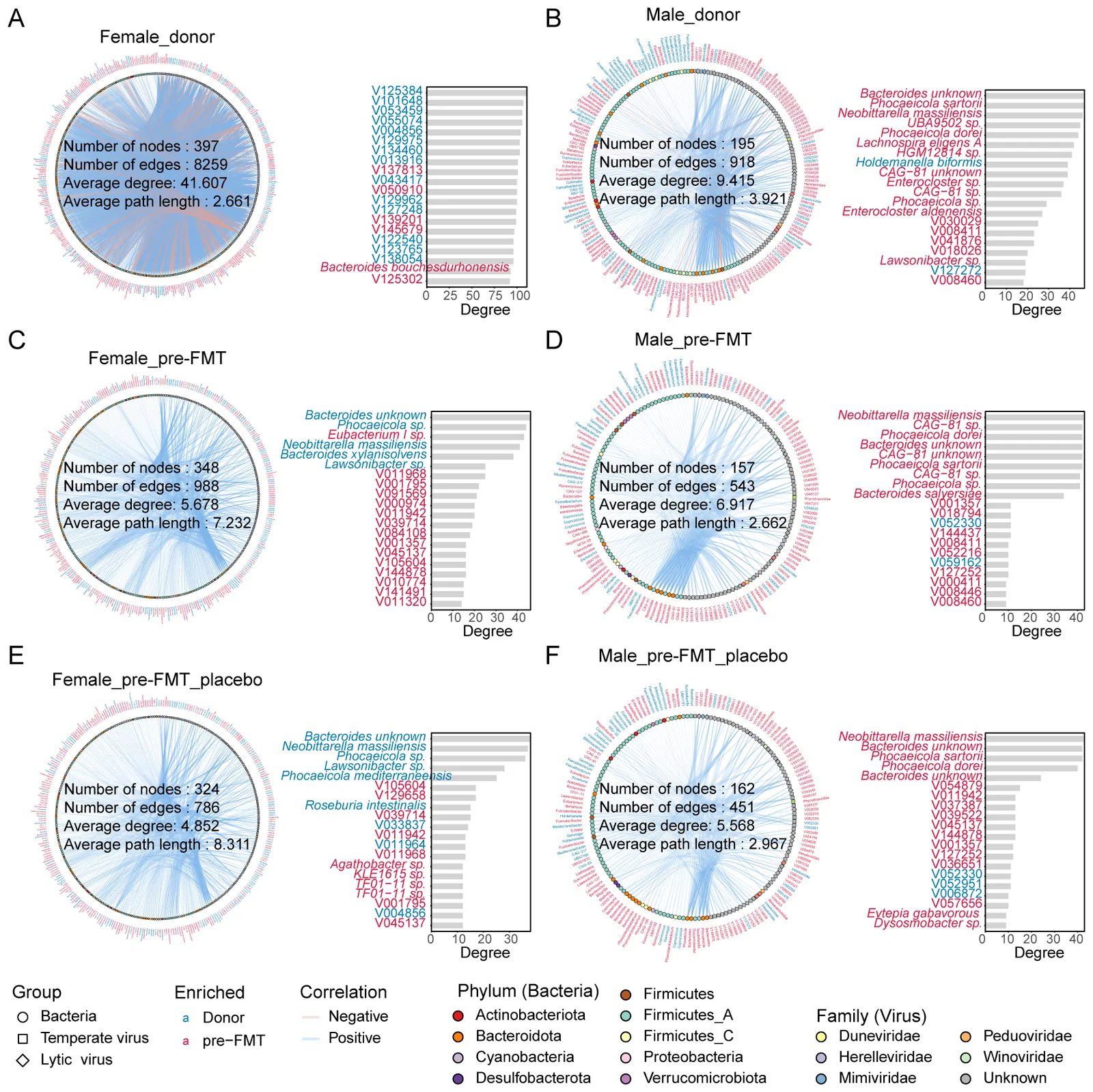
Microbial co-occurrence networks reveal sex-specific differences in gut microbiome structure between healthy donors and obese adolescents. **(A, B)** Co-occurrence networks of the gut microbiome in female **(A)** and male **(B)** healthy donors. Nodes represent microbial species (bacteria and viruses), with size proportional to degree (number of connections). Edges indicate significant positive or negative correlations (Spearman’s r > |0.4|, q-value < 0.05). The outer bar chart shows the degree distribution. **(C, D)** Pre-FMT microbial networks in female **(C)** and male **(D)** obese adolescents. **(E, F)** Pre-FMT microbial networks in placebo groups of female **(E)** and male **(F)** participants.

The analysis of microbial co-occurrence networks provides additional support for the above classification (**Figure 4**; **Supplementary Table 4**). The gut microbiota of healthy donors was characterized by a more integrated and efficient network topology. Female donor networks exhibited a high average degree (41.6), indicating dense interconnectivity, and were dominated by several high-degree hub species, most of which were viruses and a bacterium (*Bacteroides bouchesdurhonensis*) (**Figure 4A**). Male donor networks (**Figure 4B**), while having a lower average degree (9.4) than females, were still significantly more interconnected than recipient networks and featured distinct hub species (e.g., *Bacteroides unknown*, *Phocaeicola sartorii*, *Neobitarella massiliensis*). In contrast, the pre-FMT gut microbiota of obese adolescents displayed a fragmented and inefficient network structure (**Figures 4C, D**). Female recipient networks had a low average degree (5.7) and a long average path length (7.2). Although male recipient networks showed a slightly higher average degree (6.9), they similarly lacked a clearly defined, stable hub structure, potentially indicative of dysbiosis. Placebo group networks shared similar sparse and inefficient characteristics (**Figures 4E, F**). Notably, viruses were integrated into these networks, forming negative associations with specific bacterial taxa in donors. In pre-FMT networks, viruses showed positive associations with multiple bacteria, suggesting a different regulatory role. This suggesting phages as potential modulators of bacterial interaction networks.

Post-FMT network evolution was strongly sex-specific (**Supplementary Table 4**). Female recipients exhibited progressive and sustained remodeling, achieving significantly higher connectivity (average degree increased from 5.68 to 7.73) and efficiency (path length decreased from 7.23 to 6.74) by day 182. In contrast, male recipients showed only a transient improvement at day 84, with network parameters reverting toward pre-FMT by the end of the study, indicating a failure to maintain the restructured state. Viruses were integrated into these networks, forming distinct association patterns with bacteria that differed between health and disease states.

These findings demonstrate that a healthy gut microbiome is characterized by a hub-driven, interconnected network topology, which is disrupted in obesity. FMT may induce an early yet transient partial restoration of this network structure.

### 3.6 Gut microbial signatures enableprediction of obesity status

**Figure 5:**
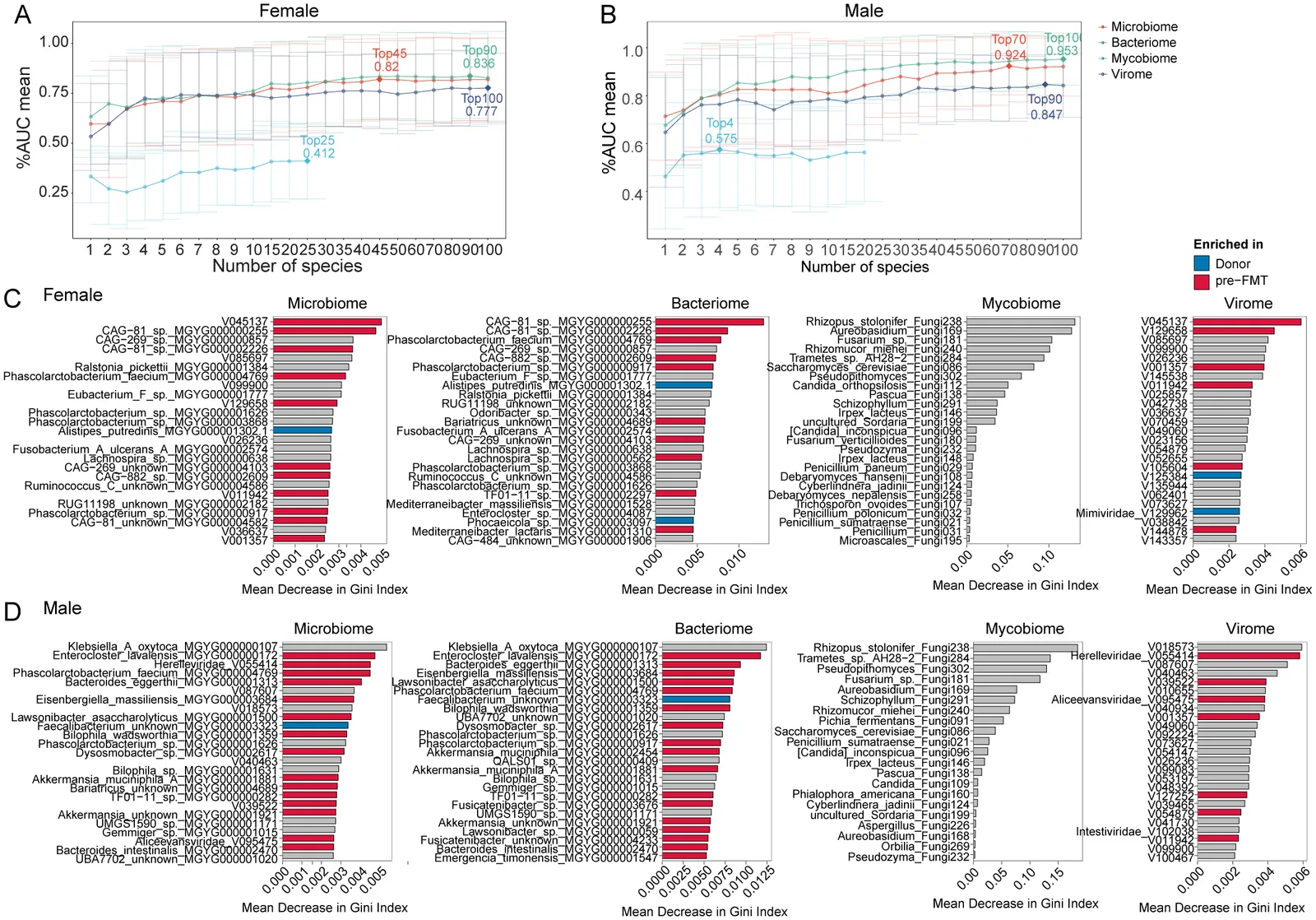
Machine learning-based classification of obesity status using gut microbial features. **(A)** Performance of multi-kingdom (Microbiome, Bacteriome, Mycobiome, Virome) models in female participants across different numbers of selected features. **(B)** Performance of multi-kingdom models in male participants. **(C)** Top microbial predictors for obesity in females, with bar colors indicating enrichment direction (blue: Donor-enriched; red: pre-FMT-enriched; black: not significantly enriched). **(D)** Top microbial predictors for obesity in males. All models were evaluated using 10-times repeated threefold cross-validation with subject-level splitting to prevent data leakage. Error bars represent standard deviation across cross-validation folds.

Machine learning models utilizing gut microbial composition achieved robust accuracy in distinguishing obesity status, with distinct performance and predictive features across sexes and microbial kingdoms (**Figure 5**). The predictive performance, as measured by the mean area under the curve (AUC) from cross-validation, varied significantly across microbial categories. In female participants (**Figure 5A**), the Bacteriome model demonstrated the highest predictive power, peaking at a mean AUC of 0.84 using the top 90 features, followed by the Multi-kingdom model (mean AUC 0.82 at Top 45). Viral features also displayed considerable predictive capacity, achieving a mean AUC of 0.78 at Top 100. In contrast, the Mycobiome alone showed relatively lower predictive capacity, reaching its maximum mean AUC of 0.41 with the top 25 features. In male participants (**Figure 5B**), similar superiority of bacterial features was observed, with the Bacteriome and Multi-kingdom models achieving peak AUCs of 0.95 (Top 100) and 0.92 (Top 70), respectively. The virome model also performed notably well in males, reaching a mean AUC of 0.85 at Top 90, while the mycobiome model plateaued earlier at a moderate mean AUC of 0.58 with only the top 4 features.

Beyond overall AUC, the classification models exhibited strong sensitivity and specificity, though performance trends diverged between sexes (**Supplementary Figure 5**). Accuracy, sensitivity, and specificity consistently improved as the number of features increased. Notably, the Multi-kingdom models achieved the highest and most stable performance across all metrics, followed by the Bacteriome models. In contrast, the Mycobiome models exhibited markedly lower sensitivity and specificity in both sexes, underscoring the limited predictive power of fungal data in this context.

Feature importance analysis further identified sex-specific gut microbial drivers of obesity. In females (**Figure 5C**), the pre-FMT enriched bacteria—such as *Ralstonia pickettii*, *Phascolarctobacterium faecium*, and *Alistipes putredinis*—along with specific viruses, emerged as strong predictors of obesity. In contrast, male obesity was primarily associated with potentially pro-inflammatory or pathobiont taxa (**Figure 5D**), including *Klebsiella oxytoca*, *Enterocloster lavalensis*, *Phascolarctobacterium faecium*, *Bacteroides eggerthii*, and *Eisenbergiella massiliensis*.

Collectively, our results demonstrate that although the exact microbial signatures vary by sex, a powerful and parsimonious signal for obesity status exists within the gut bacteriome and multi-kingdom microbial network. Fungi appear to play secondary, supportive roles rather than serving as primary classification drivers.

## 4 Discussion

Our longitudinal multi-kingdom analysis indicates that FMT in adolescents with obesity may induces a measurable yet limited and donor-dependent ecological perturbation, rather than a definitive and sustained remodeling of the gut ecosystem. Although the overall outcomes are constrained by colonization resistance and donor efficacy, our analysis extends beyond merely describing changes in microbial composition. We further evaluated the disturbance patterns and intrinsic laws of FMT on the gut ecosystem from both the taxonomic perspective of multi-kingdom microorganisms (bacteria, fungi, and viruses) and the systems ecology perspective revealed through interaction networks.

Colonization resistance and donor efficacy jointly constrain the ecological remodeling effect of FMT. The limited engraftment of donor-specific strains at the species level indicates a high degree of stability and “colonization resistance” within the host’s gut microbial community (34). Despite the introduction of a substantial exogenous microbiota via FMT, recipient-native core species, such as the short-chain fatty acid-producing *Agathobacter rectalis*, rapidly re-established dominance (**Figure 1**). This finding is consistent with the theory of inherent ecological resilience in microbial communities and may explain why metabolic benefits often attenuate over time in many FMT clinical studies. Notably, the role of *Agathobacter* in obesity was complex in the existing literature. For instance, while some studies have reported an association between increased abundance of butyrate-producing bacteria and obesity (35), others have observed a significant decrease in *Agathobacter* relative abundance in obese women following caloric restriction (36). These seemingly contradictory results suggest that the functional significance and abundance of specific microbial taxa were highly dependent on the specific metabolic context, host state, and research methodologies, highlighting the non-linear and context-dependent nature of the relationship between gut microbiota and obesity.

Furthermore, alpha-diversity analysis of the entire microbial community (bacteria, fungi, viruses) revealed no significant global shifts following FMT (**Figure 1B**), with only transient and non-significant fluctuations in the Shannon index observed in both female and male recipients. This finding contributes to the ongoing debate regarding microbial diversity and obesity—some studies report no significant difference in diversity between obese and non-obese individuals (37–39), while others indicate reduced (40–42) or even increased diversity in obesity (43, 44). Our results add new evidence, suggesting that a single course of FMT is insufficient to systematically alter the overall richness and evenness of the gut microbiota in adolescents with obesity. Dysbiosis may manifest more through changes in specific taxa or functional pathways rather than simple shifts in diversity.

Beta-diversity analysis based on Bray-Curtis distances further revealed that FMT only transiently shifted the gut bacterial community structure of recipients toward a donor-like state, after which the microbiota of most recipients, particularly males, gradually reverted to the pre-intervention state (**Figure 2**). This dynamic process reaffirms the powerful restorative capacity of a mature gut ecosystem. More critically, our study found that the extent and durability of this shift strongly depended on the donor source (**Supplementary Figure 1**). We identified specific “ high-efficacy donor batches” (e.g., DF16 for females, DM08 for males) associated with better outcomes, whereas other donors could induce initial ecological disturbance or prove ineffective. This discovery was consistent with prior research conclusions (25) and lends support to the “super-donor” hypothesis. It indicates that the success of FMT depends not merely on microbial transfer, but on the core ecological and functional properties of the donor microbiota itself—such as the presence of keystone species, community stability, functional redundancy, and interaction networks—which determine its engraftment potential and stability in the new host environment (45).

Differential multi-kingdom microbial responses and donor-driven ecological network remodeling. Building on the established limitation of overall ecological remodeling, our multi-kingdom analysis reveals a clear hierarchy in microbial responses to FMT, with all responses being modulated by donor characteristics. Our data indicate that bacteria were the primary responders to FMT, with their compositional shifts forming the core of the intervention’s ecological effect. Differential abundance analysis further elucidated this process, revealing donor- and sex-specific colonization patterns. In female recipients, donor-enriched taxa such as *Prevotella* increased initially after intervention but gradually decreased thereafter, while recipient-enriched taxa including various *Lachnospiraceae* exhibited strong resilience with limited changes in relative abundance. In male recipients, donor-enriched taxa such as *Faecalibacterium* also showed transient enrichment, whereas recipient-enriched taxa, including certain Bacteroides and *Lachnospiraceae*, gradually declined following FMT. Previous studies have also shown that *Prevotella* and *Bacteroides* were significantly increased in obese individuals, while *Faecalibacterium* was significantly reduced (46). Notably, these compositional shifts were most pronounced and sustained in recipients of high-efficacy donor batches, supporting that donor characteristics critically shape the extent and durability of bacterial community restructuring. These findings were consistent with the established dominant role of bacteria in FMT-mediated effects (47, 48). In contrast, fungal communities showed minimal change, and viral communities exhibited a distinct, transient dynamic characterized by a post-FMT increase in the proportion of lytic phages (**Supplementary Figure 4**). This aligns with observations of altered phage ecology in other dysbiotic states (49, 50) and suggests that donor-derived phages may actively participate in an initial ecological perturbation, potentially facilitating donor bacterial engraftment (51). The transient nature of this virome shift further underscores the resilience of the gut ecosystem (52).

Microbial co-occurrence network analysis provided system-level insights (**Figure 4**). Healthy donor microbiota displayed highly integrated, hub-driven networks, indicative of functional robustness. Conversely, pre-FMT recipient networks were fragmented and inefficient, a hallmark of dysbiosis consistent with findings in other obese cohorts (53). Notably, viruses were integral components of these networks, and their association patterns with bacteria differed starkly between the structured donor networks and the dysregulated recipient networks. This highlights the virome’s role as a context-dependent modulator of microbial community stability.

Prediction of obesity status by microbial signatures and clinical implications. Echoing the dynamic changes induced by FMT, pre-FMT gut microbial composition demonstrated robust diagnostic potential. Our machine learning models successfully discriminated obesity status using gut microbial features, with predictive performances varying across microbial kingdoms. The multi-kingdom and bacteriome models delivered the highest and most stable accuracies across both sexes (**Figure 5**; **Supplementary Figure 5**). In contrast, viral and fungal models exhibited substantially lower predictive capacities, underscoring the dominant role of the gut bacteriome and its synergistic interactions with other microbial realms. Furthermore, sensitivity and specificity metrics revealed that multi-kingdom approaches consistently outperformed single-kingdom models, reinforcing the utility of integrating cross-domain microbial information (**Supplementary Figure 5**). In terms of specific drivers, sex-specific microbial signatures were evident. Obesity in females was closely associated with taxa such as *Phascolarctobacterium faecium* and *Ralstonia pickettii*—the former linked to non-obese adults (54) and the latter to obesity (55). In contrast, male obesity was strongly associated with potentially pro-inflammatory or pathogenic taxa, including *Klebsiella oxytoca*, *Enterocloster lavalensis*, *Bilophila wadsworthia*, and *Eisenbergiella massiliensis* (56–58). Collectively, these findings indicate that while females and males share a similar obese phenotype, they appear to reach this state through distinct, sex-specific microbial ecological pathways.

Several limitations should be acknowledged. First, the multi-donor pooling design precludes attribution of effects to individual donors. Second, species-level profiling lacks strain resolution to track strain origins within mixtures. Third, the absence of capsule mixture sequencing prevented quantification of each donor’s relative contribution. Fourth, the single-cohort design limits generalizability to other populations given the effects of ethnicity, diet, and geography. Fifth, the machine learning models lack external validation; therefore, the reported AUCs may be optimistic and require independent confirmation. Sixth, as an exploratory study, our findings are hypothesis-generating and should be interpreted with caution. Despite these constraints, our results provide a basis for future optimization of donor selection strategies in adolescent obesity.

## 5 Conclusion

In conclusion, this study through longitudinal multi-kingdom analysis, revealed that the impact of FMT on the gut microbiota of adolescents with obesity is limited and highly donor-dependent. Bacteria served as the primary responders, while viral community changes were transient and fungal responses were minimal. FMT partially restored the “fragmented” microbial network associated with obesity. Furthermore, machine learning models based on a selected set of microbial features demonstrated robust discriminatory power for obesity status, highlighting the existence of distinct microbial ecotypes across sexes. These findings provide a theoretical foundation for the development of precision diagnostics and personalized FMT therapies.

## Data Availability

The original contributions presented in the study are included in the article/**Supplementary Material**. Further inquiries can be directed to the corresponding authors.

## Supplementary material

Supplementary Figure 1 Inter-donor variation in microbial diversity across multi-kingdom communities in female and male donors. **(A)** Box plots showing the observed diversity index (obs diversity) and Shannon diversity index for the total microbiome, bacteriome, mycobiome, and virome among four female donors (DF12, DF14, DF16, DF17). **(B)** Similar analysis for five male donors (DM03, DM05, DM07, DM08, DM12).

Supplementary Figure 2 Donor batch-specific engraftment dynamics in female recipients across multi-kingdom microbiomes. Box plots showing the average Bray-Curtis distance to donor microbiome for each microbial domain—total microbiome, bacteriome, mycobiome, and virome—in female recipients receiving FMT from four different female donors (DF12, DF14, DF16, DF17). Data are stratified by time point: pre-FMT_0_female_FMT, post-FMT_42_female_FMT, post-FMT_84_female_FMT, post-FMT_182_female_FMT, and corresponding placebo groups.

Supplementary Figure 3 Donor batch-specific engraftment dynamics in male recipients across multi-kingdom microbiomes. Box plots showing the average Bray-Curtis distance to donor microbiome for each microbial domain—total microbiome, bacteriome, mycobiome, and virome—in male recipients receiving FMT from four different female donors (DM03, DM05, DM07, DM08, DM12). Data are stratified by time point: pre-FMT_0_male_FMT, post-FMT_42_male_FMT, post-FMT_84_male_FMT, post-FMT_182_male_FMT, and corresponding placebo groups.

Supplementary Figure 4 Dynamics of temperate and lytic viruses in the gut virome following FMT in female and male adolescents. **(A)** Virome composition in females across donor, FMT recipient, and placebo groups at multiple time points (pre-FMT_0 to post-FMT_182). Left panel: Proportion of temperate (blue) and lytic (red) viruses in each group. Middle panel: Relative abundance of temperate viruses (%). Right panel: Relative abundance of lytic viruses (%). **(B)** Equivalent analysis in males.

Supplementary Figure 5 Performance metrics of the random forest models across different numbers of top microbial features (TopN) in female and male subjects. The panels show the **(A)** Accuracy, **(B)** Sensitivity, and **(C)** Specificity for female (left column) and male (right column) cohorts.
