# Supplementary material for: Fecal microbiota transplantation in adolescents with obesity induces limited and donor-dependent remodeling of the gut microbiome
Source: Front Endocrinol (Lausanne). 2026 Sep 9;17:1927870. doi: 10.3389/fendo.2026.1927870 (PMC13597358; doi:10.3389/fendo.2026.1927870)
Supplement: Supplementary file 1 [file Supplementaryfile1.docx]

**Fig. S1 Inter-donor variation in microbial diversity across multi-kingdom communities in female and male donors. (A)** Box plots showing the observed diversity index (obs diversity) and Shannon diversity index for the total microbiome, bacteriome, mycobiome, and virome among four female donors (DF12, DF14, DF16, DF17). **(B)** Similar analysis for five male donors (DM03, DM05, DM07, DM08, DM12).

**Fig S2. Donor batch-specific engraftment dynamics in female recipients across multi-kingdom microbiomes.** Box plots showing the average Bray-Curtis distance to donor microbiome for each microbial domain—total microbiome, bacteriome, mycobiome, and virome—in female recipients receiving FMT from four different female donors (DF12, DF14, DF16, DF17). Data are stratified by time point: pre-FMT_0_female_FMT, post-FMT_42_female_FMT, post-FMT_84_female_FMT, post-FMT_182_female_FMT, and corresponding placebo groups.

**Fig S3. Donor batch-specific engraftment dynamics in male recipients across multi-kingdom microbiomes.** Box plots showing the average Bray-Curtis distance to donor microbiome for each microbial domain—total microbiome, bacteriome, mycobiome, and virome—in male recipients receiving FMT from four different female donors (DM03, DM05, DM07, DM08, DM12). Data are stratified by time point: pre-FMT_0_male_FMT, post-FMT_42_male_FMT, post-FMT_84_male_FMT, post-FMT_182_male_FMT, and corresponding placebo groups.

**Fig. S4 Dynamics of temperate and lytic viruses in the gut virome following FMT in female and male adolescents. (A)** Virome composition in females across donor, FMT recipient, and placebo groups at multiple time points (pre-FMT_0 to post-FMT_182). Left panel: Proportion of temperate (blue) and lytic (red) viruses in each group. Middle panel: Relative abundance of temperate viruses (%). Right panel: Relative abundance of lytic viruses (%). **(B)** Equivalent analysis in males.

**Fig. S5 Performance metrics of the random forest models across different numbers of top microbial features (TopN) in female and male subjects.** The panels show the **(A)** Accuracy, **(B)** Sensitivity, and **(C)** Specificity for female (left column) and male (right column) cohorts.
